# Comparison of mitochondrial genome and development of specific PCR primers for identifying two scuticociliates, *Pseudocohnilembus persalinus* and *Uronema marinum*

**DOI:** 10.1186/s13071-021-04821-3

**Published:** 2021-06-10

**Authors:** Yu-Xi Huang, Sen Wang, Yan-Qi Gao, Jie-Hu Chen, Xiu-Li Wang, Rui-Jun Li

**Affiliations:** 1 Liaoning Key Laboratory of Marine Animal Immunology, Dalian Key Laboratory of Marine Animal Disease Control and Prevention, College of Fisheries and Life Science, Dalian Ocean University, Dalian, Liaoning 116023 People’s Republic of China; 2 Science Corporation of Gene, Guangzhou, Guangzhou 510000 People’s Republic of China

**Keywords:** *Pseudocohnilembus persalinus*, *Uronema marinum*, Genome comparison, Specific PCR primers

## Abstract

**Background:**

*Pseudocohnilembus persalinus* and *Uronema marinum* (Ciliophora, Scuticociliatia), as parasitic scuticociliatid ciliates, were isolated from *Scophthalmus maximus* and *Takifugu rubripes*, respectively, in our previous studies. These ciliates are morphologically very similar; hence, it is difficult to identify specific scuticociliate species using traditional classification methods for performing taxonomic research and disease control studies.

**Methods:**

We annotated the mitochondrial genomes of these two scuticociliates on the basis of previous sequencing, including analyses of nucleotide composition, codon usage, Ka/Ks, and p-distance. We also compared the nucleotide and amino acid similarity of the mitochondrial genomes of *P. persalinus*, *U. marinum*, and other 12 related ciliates, and a phylogenetic tree was constructed using 16 common genes. We chose the *nad4* and *nad7* genes to design specific PCR primers for identification.

**Results:**

*P. persalinus* and *U. marinum* were found to have a close evolutionary relationship. Although codon preferences were similar, differences were observed in the usage of codons such as CGA, CGC, and GTC. Both Ka/Ks and p-distance were less than 1. Except for *yejR*, *ymf57*, *ymf67*, and *ymf75*, the amino acid sequence similarity between *P. persalinus* and *U. marinum* was greater than 50%.

**Conclusions:**

The mitochondrial genomes of *P. persalinus* and *U. marinum* were thoroughly compared to provide a reference for disease prevention and control. The specific PCR primers enabled us to identify *P. persalinus* and *U. marinum* rapidly and accurately at the molecular level, thus providing a basis for classification and identification.

**Graphic abstract:**

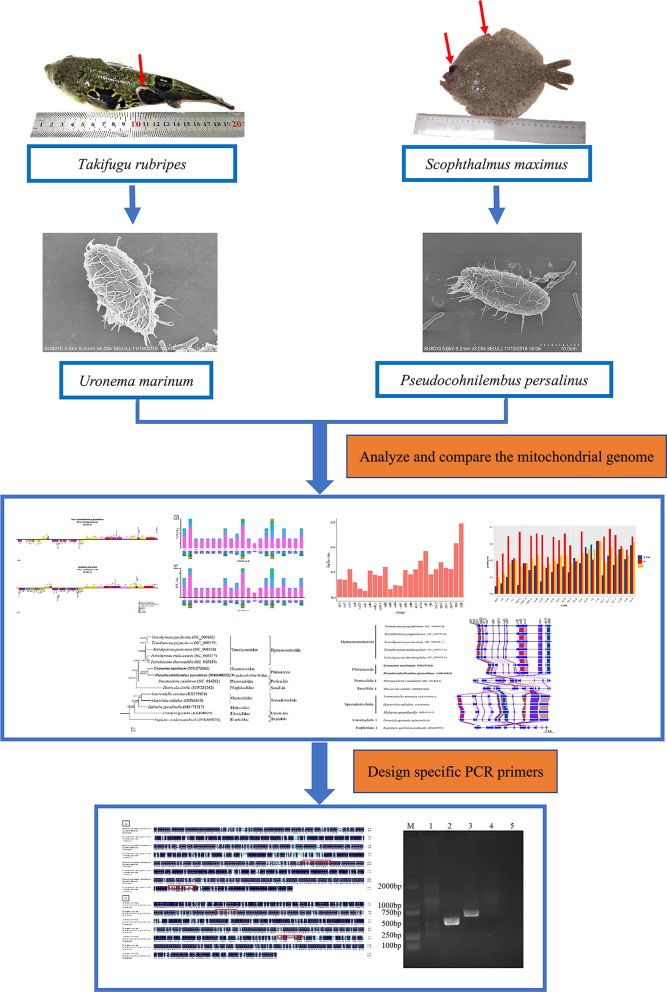

**Supplementary Information:**

The online version contains supplementary material available at 10.1186/s13071-021-04821-3.

## Background

In recent years, there has been an increase in the occurrence and extent of damage due to diseases of mariculture animals caused by ciliates. Scuticociliatosis, a disease caused by a type of ciliates known as scuticociliates, occurs globally and has led to a high fatality rate of fishes [1, 2]. Scuticociliates include approximately 20 species that cause scuticociliatosis, including *Miamiensis avidus*, *Uronema marinum*, *Uronema nigricans*, *Metanophrys orientalis*, *Pseudocohnilembus persalinus*, and *Pseudocohnilembus hargisi* [3–7]. The species of parasitic scuticociliates differ according to the different living environments of the host, but the symptoms caused by them are very similar [8]. Scuticociliates can not only invade the body surface, skin, fin, and muscle of fishes but also their abdominal cavity, kidney, pancreas, and even brain. Scuticociliates cause blackening of fish body color, tissue and organ bleeding, ulceration, and other pathological changes, leading to the death of *Paralichthys olivaceus*, *Thunnus maccoyii*, *Scophthalmus maximus*, and *Dicentrachus labrax* [4, 9–12]. Thus, to prevent and control scuticociliatosis effectively and rapidly, it is important to accurately detect scuticociliates. However, the morphological forms of the subclass Scuticociliatia are very similar. The body of scuticociliates is 20–45 μm long, melon seed-shaped, and covered with cilia. Therefore, it is difficult to distinguish scuticociliates using a light microscope. Previously, the species of scuticociliates could only be identified through repeated verification by a series of methods such as scanning electron microscopy, protein silver staining, and silver impregnation. Although the morphological analysis of scuticociliates is a useful approach to identify them, it is time-consuming and laborious, and is thus not suitable for wider use practically in treating scuticociliatosis. Moreover, morphological analysis is based on staining techniques, which may obscure subtle changes, leading to identification errors [13]. The ribosomal small subunit rDNA (SSU-rDNA) sequence analysis method is a universal tool for parasite classification. However, the SSU-rDNAs of related parasite species show few differences, which limits the identification of closely related species [14].

In a preliminary study, we isolated *P. persalinus* and *U. marinum* from *Scophthalmus maximus* and *Takifugu rubripes*, respectively [15, 16]. *P. persalinus* and *U. marinum* are dominant scuticociliate pathogens that are distributed in the north of China marine aquaculture area [17]. In our previous studies, we sequenced the whole mitochondrial genomes of *P. persalinus* and *U. marinum* [15, 16]. In the present study, we analyzed interspecific differences in the mitochondrial genome between *P. persalinus* and *U. marinum* by comparing codon usage, Ka/Ks, and p-distance. We also revealed evolutionary relationships and mitochondrial genome differences between these species and 12 related ciliates by constructing a phylogenetic tree and comparing nucleotide and amino acid similarity of the mitochondrial genomes. Specific PCR primers for identification were designed according to the specific genes in the mitochondrial genomes of *P. persalinus* and *U. marinum*. These primers enable easy, rapid, and accurate species identification at the molecular level. The use of PCR primers to identify scuticociliates provided the basis for the classification and identification of scuticociliates.

## Methods

### Whole mitochondrial genomes of *P. persalinus* and *U. marinum*

The whole mitochondrial genome sequences of *P. persalinus* and *U. marinum* were deposited in NCBI (https://www.ncbi.nlm.nih.gov/) with accession numbers MH608212 and MG272262, respectively.

### Annotation of the mitochondrial genome

The MITOS (http://mitos.bioinf.uni-leipzig.de/index.py) [18] and ORF Finder (https://www.ncbi.nlm.nih.gov/orffinder/) tools were used to preliminarily annotate mitochondrial genome sequences. BLASTp and BLAST (https://blast.ncbi.nlm.nih.gov/Blast.cgi) were used to compare the preliminary results of annotation with encoded protein and rRNA sequences belonging to the reported related species in order to verify the accuracy of the results and make corrections. The schematic diagram was drawn using OGDRAW (https://chlorobox.mpimp-golm.mpg.de/OGDraw.html).

### Analysis of codon usage

The codon usage bias of *P. persalinus* and *U. marinum* was compared. The RSCU was calculated using the formula described in the study by Sharp and Li [19].

### Analysis of Ka/Ks and p-distance

Ka/Ks analysis was performed on 25 common encoding genes of *P. persalinus* and *U. marinum*. MUSCLE v3.8.31 (http://www.drive5.com/muscle/) software was used to compare the selected 25 common encoding genes of *P. persalinus* and *U. marinum* [20]. The KaKs_Calculator 2.0 was then used to calculate the Ka/Ks of all compared sequences [21], and MEGA6 software was used to calculate p-distance [22].

### Phylogenetic analysis

The complete mitochondrial sequences of 12 ciliates related to *P. persalinus* and *U. marinum* were downloaded from NCBI for phylogenetic tree construction. The sequences used for comparison and their GenBank accession numbers were as follows: *Tetrahymena paravorax* (Accession Number: NC_008338.1), *Tetrahymena pyriformis* (Accession Number: NC_000862.1), *Tetrahymena thermophila* (Accession Number: NC_003029.1), *Paramecium caudatum* (Accession Number: NC_014262.1), *Tetrahymena pigmentosa* (Accession Number: NC_008339.1), *Tetrahymena malaccensis* (Accession Number: NC_008337.1), *Laurentiella strenua* (Accession Number: KX529838.1), *Thuricola similis* (Accession Number: MW221262), *Oxytricha trifallax* (Accession Number: JN383843), *Halteria grandinella* (Accession Number: MT471317), *Euplotes vanleeuwenhoeki* (Accession Number: MK889230), and *Urostyla grandis* (Accession Number: KX494929.1). Sixteen common encoding genes of the complete mitochondrial genome of all ciliates were selected for phylogenetic tree construction. MUSCLE v3.8.31 software (http://www.drive5.com/muscle/) was used to compare individual genes among all 14 ciliates. The jModelTest 2.1.7 (https://code.google.com/p/jmodeltest2/) [23] was used for testing the nucleic acid model, and the model with the minimum Akaike information criterion (AIC) value was selected as the best model for phylogenetic tree construction. MrBayes v3.2.6 (http://nbisweden.github.io/MrBayes/manual.html) was used to construct phylogenetic trees with Bayesian inference [24], and RAxML 8.1.5 (https://sco.h-its.org/exelixis/web/software/raxml/index.html) was used to construct a phylogenetic tree using the maximum likelihood (ML) method, with the bootstrap value set to 1000 [25].

### Comparison of nucleotide and amino acid similarity of mitochondrial genomes of 14 related ciliates

The COGs and identity comparison of the whole mitochondrial genomes belonging to *P. persalinus*, *U. marinum*, and 12 other related ciliates were analyzed [26]. According to the species arrangement and common gene information of the phylogenetic tree, genoPlotR (http://genoplotr.r-forge.r-project.org/) [27] was used to compare and analyze the common genes of these 14 species.

### Design of specific PCR primers for identification

The whole mitochondrial genome sequences of *P. persalinus* and *U. marinum* were compared. Next, genes with both intraspecific relative conservatism and interspecific diversity among scuticociliates were selected. Among the selected genes, we chose *nad4* and *nad7*. Primer Premier 5 was used to design specific primers based on *nad4* and *nad7*. Primers P2-F (5ʹ-GTTATGGTTACAATGTTTGGTGTTAT-3ʹ) and P2-R (5ʹ-TATAGTACCAACATGTTTTCTCATCA-3ʹ) were designed based on the *nad4* sequence of *P. persalinus*; primers P3-F (5ʹ-ATTGTTCTATGCTTATGCAA-3ʹ) and P3-R (5ʹ-TGTTTAGTAGAACTATTATTCAT-3ʹ) were designed based on the *nad7* sequence of *U. marinum*. The PCR reaction system consisted of a 40 μL reaction mixture containing 20 μL KOD buffer, 0.8 μL KOD enzyme (KOD FX, Toyobo Biotech Co., Shanghai), 8 μL 2 mM dNTPs, 2 μL forward primer, 2 μL reverse primer, 2 μL model DNA, and 5.2 μL sterile purified water. The PCR reaction conditions were as follows: 94 °C for 3 min, 98 °C for 15 s, 56 °C for 30 s, 68 °C for 45 s, followed by 35 cycles and 68 °C for 5 min. Finally, the results were confirmed by agarose gel electrophoresis. The PCR products were sent to Sangon Biotech Co. (Shanghai, China) for nucleic acid sequencing, and the obtained sequences were compared with the target sequences.

## Results

### Comparison of mitochondrial genomes of *P. persalinus* and *U. marinum*

**Fig. 1:**
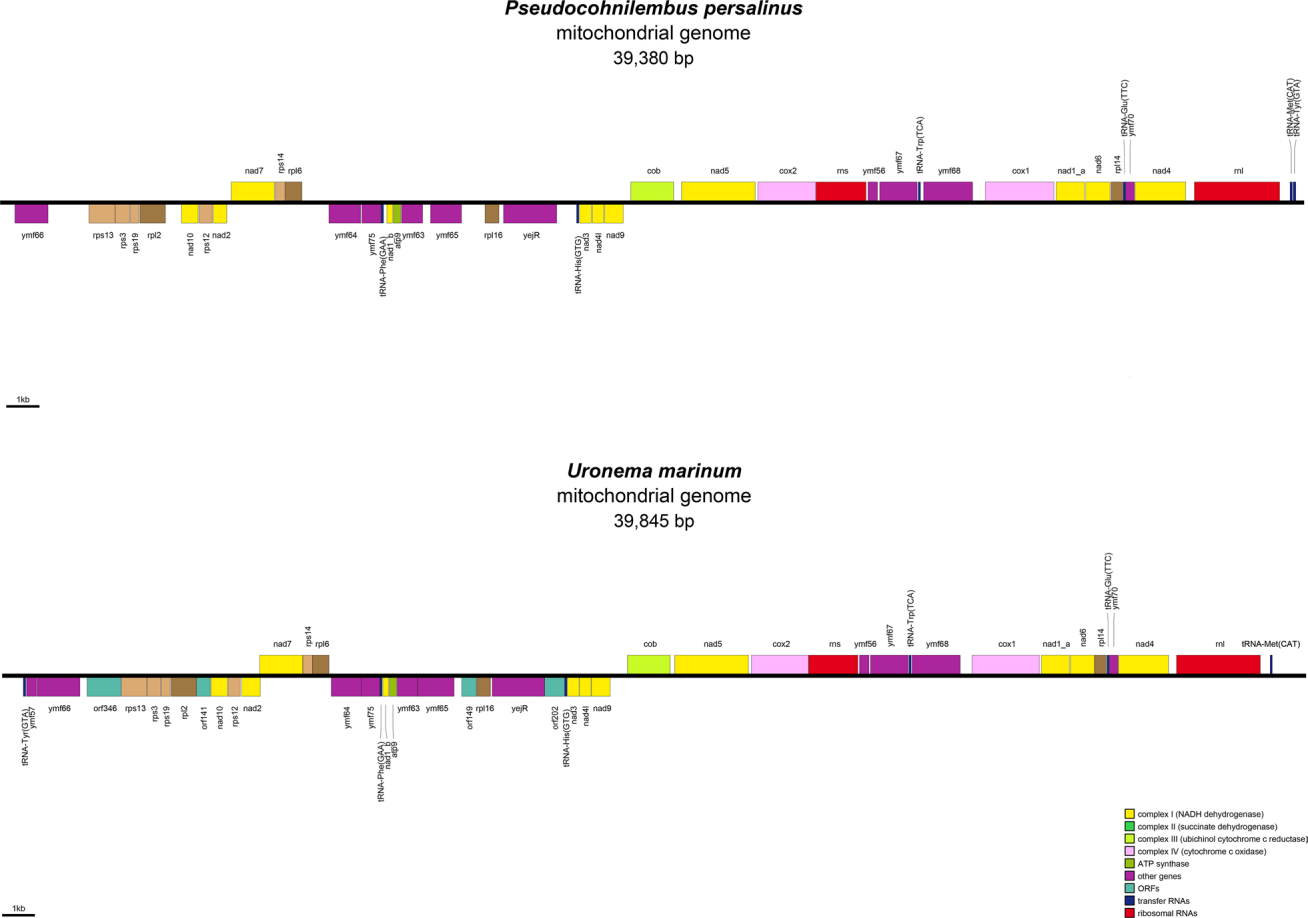
Annotation of mitochondrial genome of *P. persalinus* and *U. marinum*

According to the data given in Table 1, the entire mitochondrial genome of *U. marinum* was 1470 bp longer than that of *P. persalinus*. The protein-coding gene region of *U. marinum* was 755 bp longer than that of *P. persalinus* and contained three more genes than that of *P. persalinus*. Although both *P. persalinus* and *U. marinum* have a strong AT preference, the AT preference of *U. marinum* was stronger than that of *P. persalinus*. The AT percent in the entire mitochondrial genome of *P. persalinus* was 77.04%, and that of *U. marinum* was 80.99%. The AT (all) of *U. marinum* was 4.44% greater than that of *P. persalinus*, and the AT (third) of *U. marinum* was 9.8% greater than that of *P. persalinus*. Figure 1 shows apparent differences between the mitochondrial genomes of *P. persalinus* and *U. marinum*. The order and distribution of heavy and light chains of all common amino acid coding genes were consistent. In *U. marinum*, *ymf57*, *orf346*, *orf141*, *orf149*, and *orf202* were annotated, and the gap between *rpl6* and *ymf64*, *ymf63* and *ymf65*, and *nad9* and *cob* was larger.

**Table 1:** Comparison of mitochondrial genomes of *P. persalinus* and *U. marinum*

Species	Length (bp)	Entire genome							Protein-coding gene		
		A (bp)	T (bp)	C (bp)	G (bp)	N (bp)^a^	Genes	GC (%)	Length (aa)	AT (%) (all)	AT (%) (3rd)
*Pseudocohnilembus persalinus*	38,375	14430	15135	4101	4509	200	40	22.44	9174	77.01	76.18
*Uronema marinum*	39,845	15869	16404	4562	4010	0	43	19	9929	81.45	85.98

^a^The mitochondrial genome of *P. persalinus* had two gaps, and the length of gaps was unknown. The number of N in each gap region was in accordance with NCBI sequence submission requirements, and the default was 100

Length (aa): Total length of amino acid sequence of all mitochondrial proteins. “aa” represents amino acids; AT (%) (all): The percentage of the A and T base of the coding region sequence of all proteins in the total coding region sequence; AT (%) (3rd): The percentage of the sequence consisting of the sum of A and T bases in the third base of all amino acid codons

### Analysis of codon usage

**Fig. 2:**
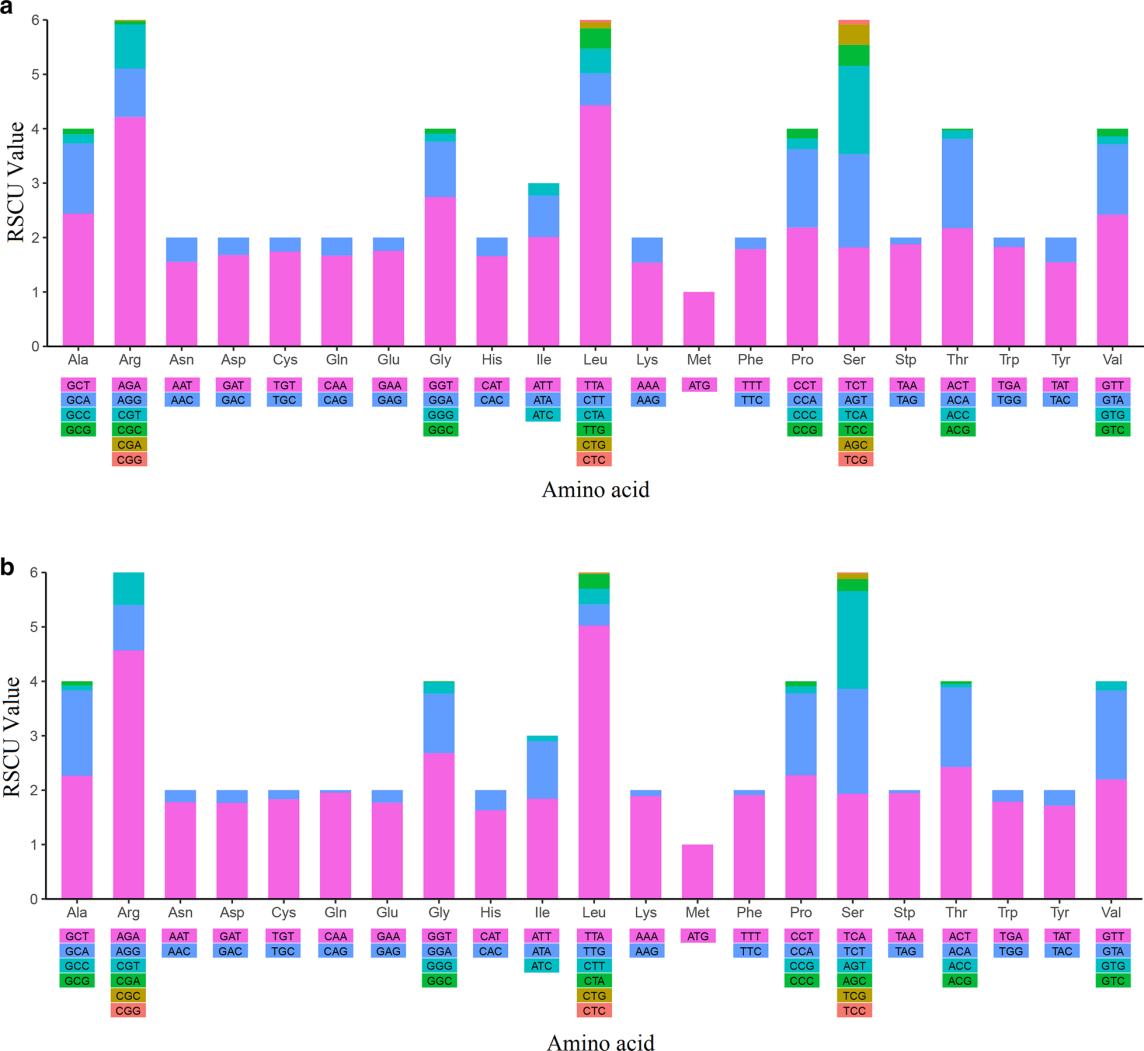
Relative synonymous codon usage of the mitochondrial genome of *P. persalinus* and *U. marinum*. **a** *P. persalinus*. **b** *U. marinum*

Figure 2 shows that in general, the whole mitochondrial genome of *P. persalinus* and *U. marinum* had almost the same preference for relative synonymous codons, with only a slight difference. The usage of CAG that coded for Gln and AAG that coded for Lys in *P. persalinus* was higher (0.286 and 0.353, respectively) than that in *U. marinum*. In *P. persalinus*, CGA and CGC coded for Arg and GTC coded for Val. The usage degrees were 0.020, 0.061, and 0.140, respectively. However, in *U. marinum*, CGA and CGC were not used to code for Arg; in contrast, the usage of AGA for coding Arg was 0.353 higher than that for *P. persalinus*; moreover, GTC was not used in *U. marinum* to code for Val.

### Analysis of Ka/Ks and p-distance

**Fig. 3:**
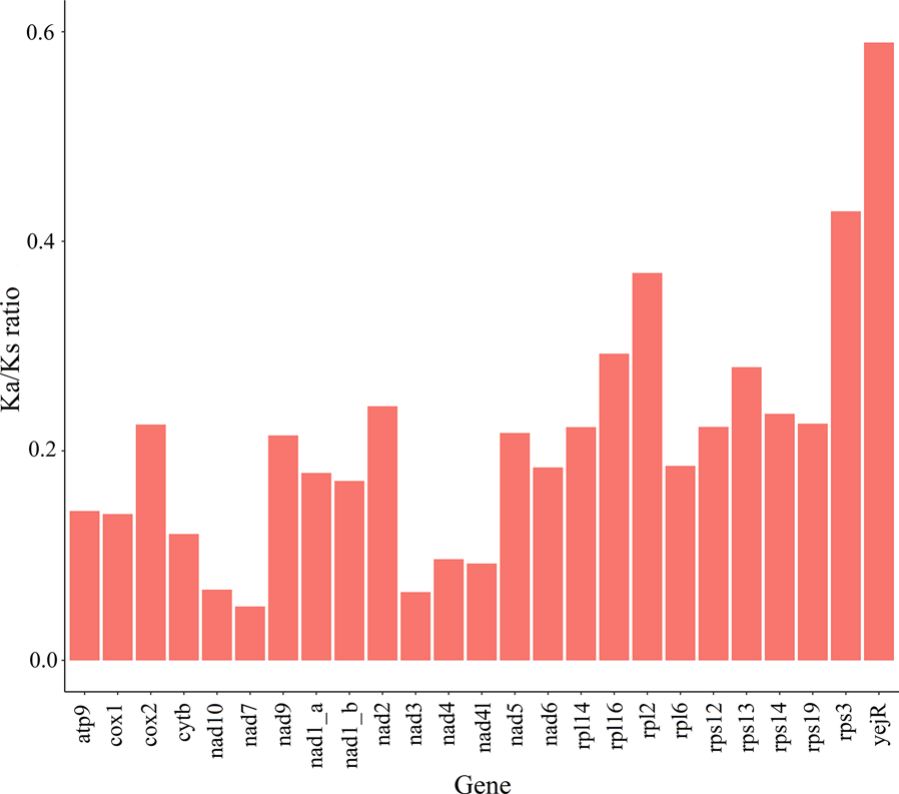
Ka/Ks analysis of 25 common encoding genes of *P. persalinus* and *U. marinum*

The Ka/Ks values (Fig. 3) of 25 common encoding genes of *P. persalinus* and *U. marinum* were less than one, which indicated that the encoding genes were mainly subjected to negative selection and the overall gene evolution tended to be conservative. Among the 25 common encoding genes, Ka/Ks of *yejR* was relatively high at 0.59, which suggested that it may have a relatively relaxed selection pressure. The specific value of Ka/Ks is shown in Additional file 1: Table S1.

**Fig. 4:**
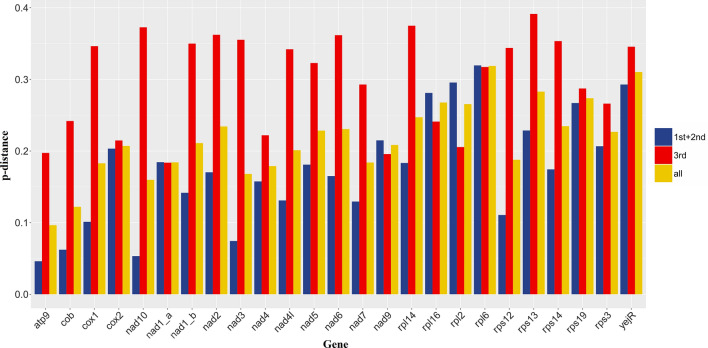
p-Distance analysis of 25 common encoding genes of *P. persalinus* and *U. marinum*. 1st + 2nd: The p-distance calculated by extracting the DNA sequence consisting of the first and second bases of the encoding gene; 3rd: The p-distance calculated by extracting the DNA sequence consisting of the third bases of the encoding gene; All: The p-distance calculated from the DNA sequence of the CDS region corresponding to the encoding gene

The data provided in Fig. 4 showed that the 3rd p-distance, except for *nad1-a*, *nad9*, *rpl16*, *rpl2*, and *rpl6*, was higher than the p-distance obtained by the other two calculation methods, and the largest difference was observed for *nad10*. The codons of the *cox2*, *nad1-a*, *nad9*, *rpl6*, and *rps19* genes did not show much difference in the p-distance calculated from the first and second bases or the third base and the CDS region, probably because Met or proteins with strong codon preference accounted for a large proportion of the proteins encoded by them.

### Phylogenetic tree

**Fig. 5:**
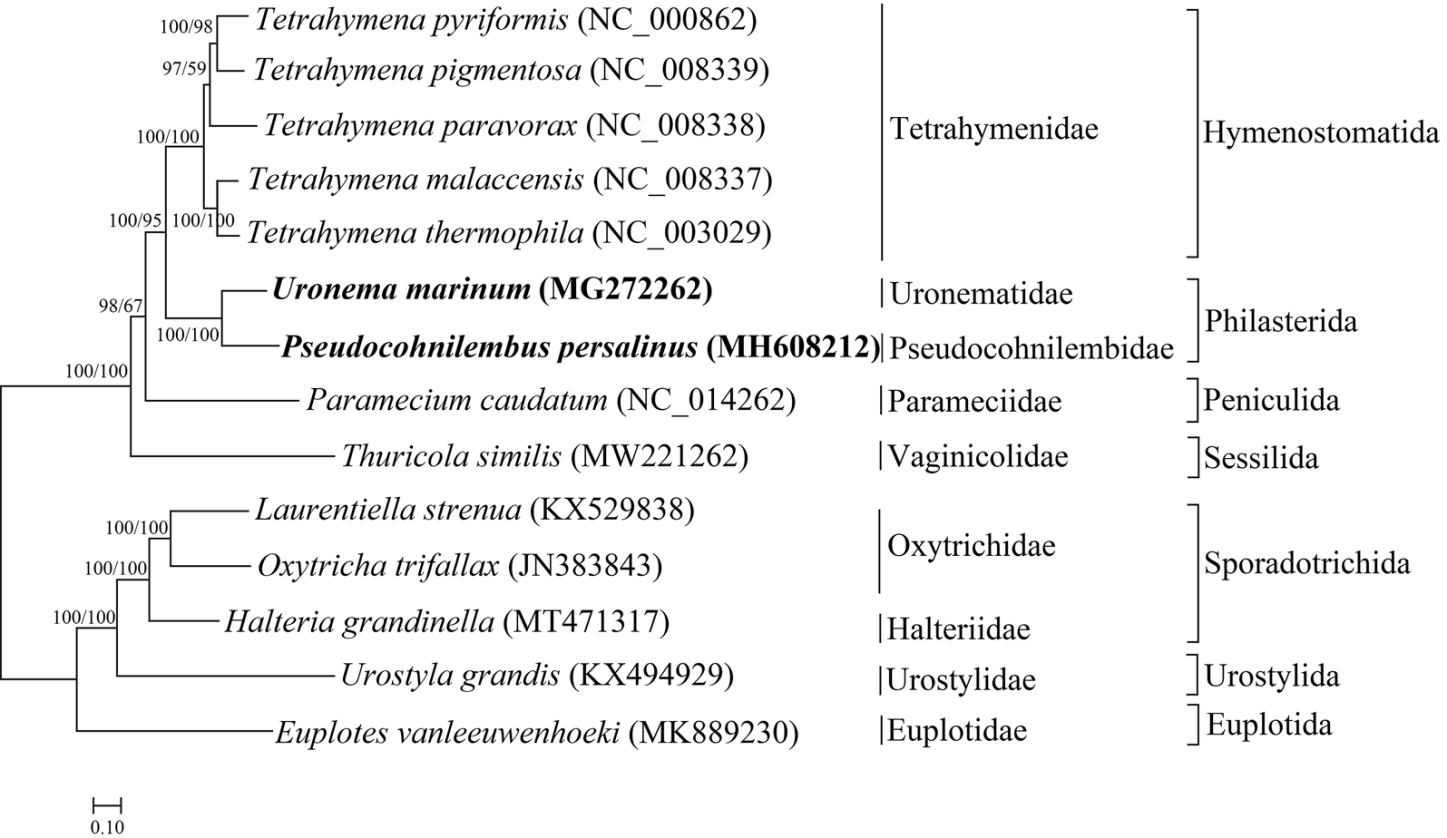
Phylogenetic tree. The length of the branches indicates the genetic distance between the ciliates. About the bootstrap value, the first value is the result of the BI algorithm, and the latter is the result of the ML algorithm

The clustering results showed that *P. persalinus* and *U. marinum* were clustered together. *P. persalinus* and *U. marinum* belonged to Philasterida and were closely related to *Paramecium caudatum* which belonged to Peniculida. Other ciliates were in obvious clades: Hymenostomatida, Peniculida, Sessilida, Sporadotrichida, Urostylida, and Euplotida (Fig. 5).

### Comparison of nucleotide and amino acid similarity of the mitochondrial genomes of 14 related ciliates

The results showed that most of the functional genes annotated by COGs were related to secondary metabolite biosynthesis, transport, and catabolism. This indicates that these functions were essential for the growth and reproduction of ciliates and that these genes were also the most conservative ones. Except for *yejR*, *ymf57*, *ymf67*, and *ymf75*, the amino acid sequence identity of other genes of both *P. persalinus* and *U. marinum* was greater than 50%. The amino acid sequence of the *nad10* gene was the most identical among the compared species; the identity of *nad10* was > 80% in six ciliates and > 60% in all ciliates. Additionally, the *rps12*, *nad7*, *nad1-b, atp9*, *nad3*, *cob*, *cox1*, *nad1-a*, and *nad4* genes were also highly identical. The identity of the *nad3* and *nad4* genes in seven ciliates; the *nad1-b, atp9*, *cob*, *cox1*, and *nad1-a* genes in eight ciliates; the *rps12* gene in nine ciliates; and the *nad7* gene in 12 ciliates was > 50%, which indicated that they were relatively conserved genes among these species. The identity of *nad7* was < 50% only in *L. strenua*. The *nad7* gene plays a role in the biosynthesis, transport, and catabolism of secondary metabolites as well as in energy production and conversion. Perhaps *L. strenua* is very different from other ciliates with regard to some of these aspects. The *ymf66*, *rps13*, *rps19*, *rpl2*, *rps14*, *rpl6*, *ymf64*, *ymf63*, *ymf65*, *rpl16*, and *ymf56* genes had a high identity of greater than 50% in *P. persalinus* and *U. marinum*, but less than 50% identity in other ciliates. This is probably because *P. persalinus* and *U. marinum* are phylogenetically close species which are isolated from other ciliates.

In addition, the identity of several nucleotide sequences such as *tRNA-Tyr* (GTA), *nad10*, *rps12*, *nad7*, *tRNA-Phe* (GAA), *nad1-b*, *atp9*, *nad3*, *cob*, *rns*, *tRNA-Trp* (TCA), *cox1*, *nad1-a*, *nad4*, and *rnl* was relatively high. This result is consistent with the identity of amino acid sequences. Among them, *rns* and *rnl* encoded rRNA; *tRNA-Tyr* (GTA), *tRNA-Phe* (GAA), and *tRNA-Trp* (TCA) encoded tRNA; *nad10*, *nad7*, *nad1-b*, *atp9*, *nad3*, *cob*, *cox1*, *nad1-a*, and *nad4* were related to the biosynthesis of secondary metabolites, transport, and catabolism as well as to energy production and conversion; and *rps12* was related to chromatin structure and dynamics as well as to the biosynthesis of secondary metabolites, transport, and catabolism. This finding showed that all 14 ciliates had similar secondary metabolite transport and metabolic patterns.

**Fig. 6:**
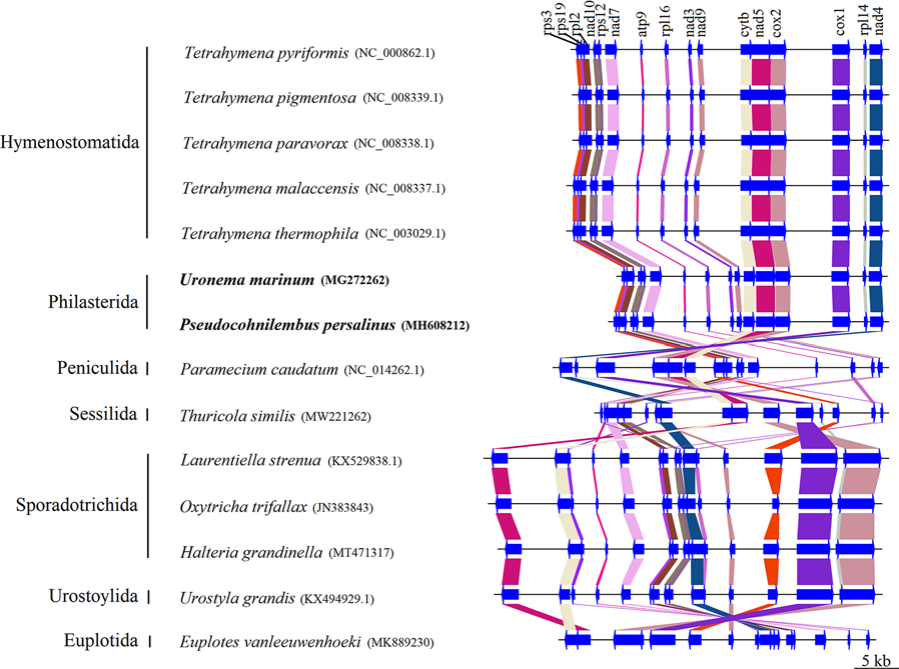
Comparison and analysis of the common genes of 14 ciliates. An arrow to the right indicates that the gene is present on the heavy chain, and an arrow to the left indicates that the gene is present on the light chain

Combined with the analysis of the phylogenetic tree (Fig. 5), it was found that the length and arrangement of the common 16 genes of these ciliates are very different at the order level (Fig. 6). Considering the order Philasterida to which *P. persalinus* and *U. marinum* belong as a benchmark, in the order Hymenostomatida, which is most closely related to Philasterida, the common genes are not different in the order in which they are arranged, with only slight differences in the length and distribution of light and heavy chains. A large number of rearrangements occurred in other orders of ciliates, wherein the length and distribution of light and heavy chains are also very different. Moreover, Sporadotrichida and Urostylida have the same sort order of common genes, wherein *rps12* is slightly distant from *nad4* in Urostylida. In all 14 ciliates, *rps19* and *rpl2*, and *nad10* and *rps12* are always next to each other.

### Specific primer design and amplification

**Fig. 7:**
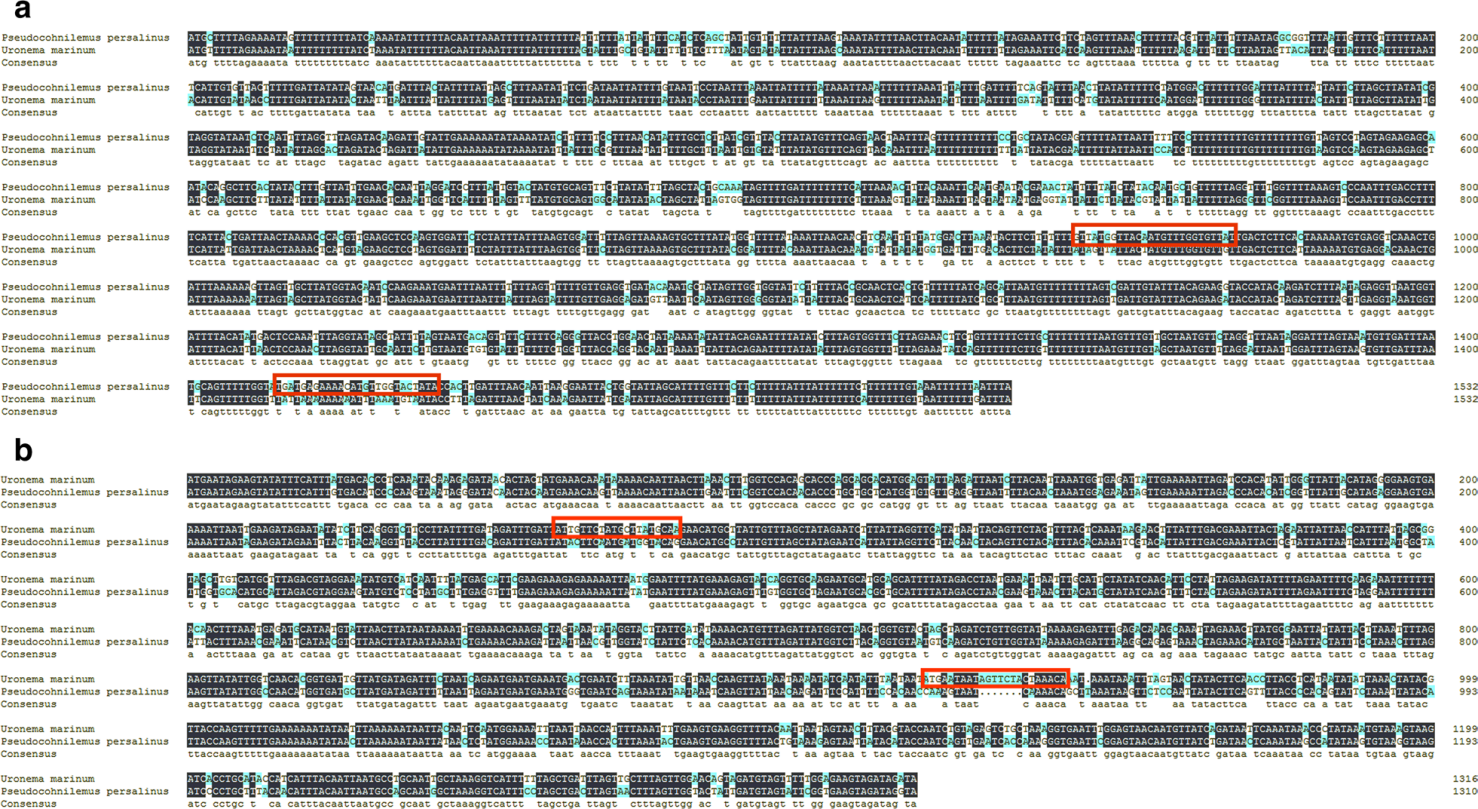
Comparison of selected gene sequences and design of primers. **a** Comparison of the *nad4* gene between *P. persalinus* and *U. marinum*. The first line is *P. persalinus* and the second one is *U. marinum*. **b** Comparison of the *nad7* gene between *P. persalinus* and *U. marinum*. The first line is *U. marinum* and the second one is *P. persalinus*. The two primers are indicated by red boxes

**Fig. 8:**
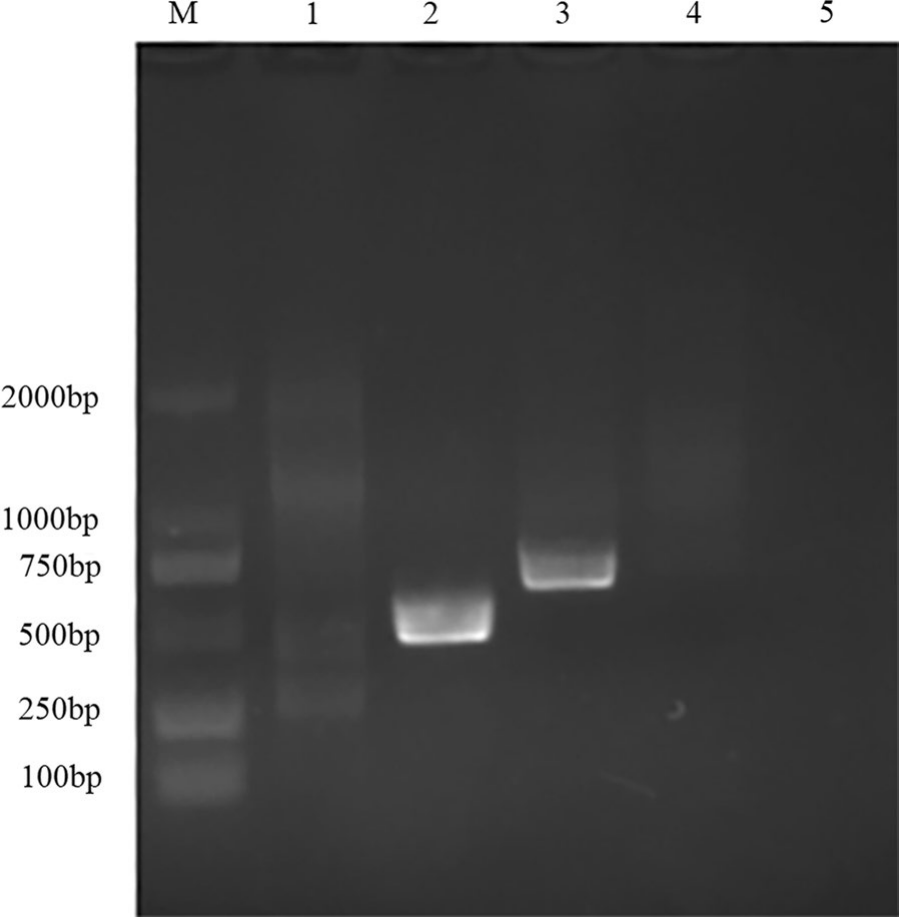
Result of specific primer amplification. M: Marker; Lane 1: The DNA template was *U. marinum* and the primer was P2; Lane 2: DNA template was *P. persalinus* and the primer was P2; Lane 3: DNA template was *U. marinum* and the primer was P3; Lane 4: DNA template was *P. persalinus* and the primer was P2; Lane 5: Blank control

The result of comparison of the selected gene sequences and design of primers is shown in Fig. 7. The amplification results of specific primers (Fig. 8) indicated that the designed specific primers had good specificity. When the P2 primer was used to amplify the DNA of *P. persalinus* and *U. marinum*, only the lanes using the *P. persalinus* gene as a template showed a single clear band. When the P3 primer was used to amplify the DNA of *P. persalinus* and *U. marinum*, only the lanes using the *U. marinum* gene as a template showed a single clear band. The result of sequence comparison showed that the amplified sequence of the specific primer was consistent with the target sequence.

## Discussion

In the present study, by performing a comparative analysis of the mitochondrial genomes of *P. persalinus* and *U. marinum*, which are very similar in morphological forms, the *nad4* and *nad7* genes were first proposed as molecular markers to differentiate these ciliate species. The interaction between nicotinamide adenine dinucleotide (NAD+) and NAD+--dependent enzyme plays an important role in cellular redox reactions, energy metabolism, and signal transduction processes. NADH dehydrogenase subunit genes include *nad1*, *nad4*, *nad2*, *nad5* [28], *nad7*, and so on. Several studies have reported the application of NADH dehydrogenase subunit genes in species identification and genetic variation as interspecific genetic markers. The *nad1* (NADH dehydrogenase subunit 1) gene has a higher nucleotide replacement rate and greater variability than other mitochondrial genes [29]. Choudhary et al. deduced the genetic structure and diversity of *Bactrocera zonata* based on the *nad1* gene and *cox1* (mitochondrial cytochrome c oxidase subunit 1) gene [30]. Mikaeili et al. used the *nad*1 and *cox1* genes to identify *Toxocara canis* and *Toxocara cati* [31]. The *nad4* (NADH dehydrogenase subunit 4) gene is one of the genes that evolved faster in the mitochondria. In the field of the classification, identification, and population inheritance of parasites, many scholars believe that the *nad4* gene sequence is an ideal genetic marker. For example, Li et al. used *nad4* to find interspecies differences among *T. canis*, *T. cati*, *Toxocara malaysiensis*, *Toxocara vitulorum*, and *Toxascaris leonina* from different regions [32]. Hao et al. found that the genetic variation of the *cox*1 gene in *Ascaridia galli* was lower than that of the *nad4* gene, and they therefore considered that the *nad4* gene was more suitable than the *cox1* gene as a molecular marker for studying the genetic variation of *A. galli* [33]. The *nad7* gene was also considered to be an accurate fragment for species identification and phylogenetic reconstruction; it was used for identifying Amoebozoa [34] and was also reported to be used for identifying Russian wild ginseng and *Mielichhoferia elongata* [35, 36]. The experimental results of the present study also proved that the *nad4* and *nad7* genes were ideal genetic markers for studying the identification of ciliate species.

The morphological forms of scuticociliates are too similar to be identified and distinguished visually. In recent years, because of the improvements in methods for obtaining molecular information of ciliates and the wide application of bioinformatics analytical methods, genetic analysis of ciliates has become a common method to study the phylogenetic evolution of ciliates. However, most gene analyses of ciliates were limited to basic gene sequencing, and the subsequent analysis was mostly limited to the analysis and comparison of SSU-rDNA and 18S rRNA. For example, Stidworthy et al. used SSU-rDNA to analyze and identify *Philasterides dicentrarchi*, which lives in sharks [37], and Felipe et al. identified *P. dicentrarchi* and *M. avidus* as different species using 18S rRNA, α-tubulin, and β-tubulin genes [38]. However, these methods cannot fully meet the requirements of ciliate classification and function research; therefore, reports on using different methods to analyze ciliate genes have gradually increased. *P. persalinus* was identified using a fluorescent dye-labeled SSU-rDNA-targeted oligonucleotide probe, which was optimized in fluorescence in situ hybridization (FISH) by Zhan et al. [39]. Xiong et al. sequenced the whole genome of *P. persalinus* and analyzed horizontal gene transfer (HGT) to understand its virulence [40]. Whang et al. found that the genetic differences in the *cox1* sequence were greater than those of SSU-rDNA in *P. persalinus*, *P. longisetus*, *U. marinum*, and *M. avidus*. The authors designed specific primers based on the *cox*1 sequence to successfully identify them [13]. In the present study, specific primers were designed based on the *nad4* gene and the *nad7* gene of *P. persalinus* and *U. marinum*. These primers could serve as a fast and accurate tool for species identification of scuticociliates. The function and composition of the mitochondrial genomes of *P. persalinus* and *U. marinum* were also analyzed in detail to provide a solid data foundation for follow-up research.

## Conclusions

In conclusion, the whole mitochondrial genomes of *P. persalinus* and *U. marinum* were thoroughly compared by analyzing nucleotide composition, codon usage, Ka/Ks, and p-distance. In addition, we compared amino acid and nucleotide identity of *P. persalinus* and *U. marinum* with 12 other related ciliates and constructed a phylogenetic tree. We have successfully designed specific PCR primers to identify *P. persalinus* and *U. marinum* using the *nad4* and *nad7* genes.

## Supplementary Information

**Additional file 1: Table S1.** Ka/Ks values of 25 common encoding genes of *P. persalinus* and *U. marinum.*

## Data Availability

The whole mitochondrial genome sequences of *P. persalinus* and *U. marinum* are deposited at NCBI (https://www.ncbi.nlm.nih.gov/), with the following accession numbers: MH608212 and MG272262. The other data supporting our findings and conclusions are available in the article.

## Abbreviations

PCR: Polymerase chain reaction
NCBI: National Center for Biotechnology Information
ORF: Open reading frame
RSCU: Relative synonymous codon usage
COGs: Clusters of Orthologous Groups
Ka: Number of non-synonymous substitutions per non-synonymous site
Ks: Number of synonymous substitutions per synonymous site
